# The National Medical Union (Official)

**Published:** 1914-03-14

**Authors:** 


### THE NATIONAL MEDICAL UNION (Official).

### Policies Favoured by Local Branches.

In response to a request for resolutions and suggestions on the subject of policy for submission to the meeting of Council, to be held on March 21, the following have been received from the Associations named, and are published for the information of members:—

### Edinburgh.

1. The policy of the National Medical Union should be the drastic amendment of the National Insurance Acts in accordance with the principles of freedom on the part of the profession and public as laid down in its policy.

2. That a National Medical Service is necessary for the genuine poor classes of the community.

### Huddersfield.

1. That this Association supports the outline of policy already put forward by the Council of the Union, together with unrestricted free choice of doctor, medical control in medical matters, and payment for work done.

2. That the Union should make every effort to secure amendment of the National Insurance Acts in the direction of substituting a voluntary for a compulsory system.

3. That the best efforts of the Union should be directed to safeguarding the interests of non-panel practitioners.

4. That the forthcoming statement of policy should be sent to every man not on the panel.

### Liverpool.

1. That the National Medical Union should concentrate its efforts on acquiring for insured persons the unhampered right of making their own arrangements with duly registered medical practitioners, as this would include free choice of doctor and lead automatically to a reduction of the income limit.

2. That the ways by which this should be attempted are: (*a*) A test case in the Law Courts on the unallotted money; (*b*) Laying our views before all members of Parliament by means of a letter on the lines of that now submitted by us to the Council; (*c*) Encouragement of the public against the Acts by official participation in all pending bye-elections.

### Manchester.

1. To secure complete freedom of doctor and patient.

2. To render every service possible to non-panel practitioners in their unequal struggle against the difficulties brought about by acceptation of service under the National Insurance Acts by panel practitioners.

3. To place before the public in any and every way possible the dangers of the Acts to public health, their injustice to doctor and patient alike, and the iniquity of continuing to work them under the present conditions.

4. To prepare amendments to the Acts, with the view of advising any legislative authority if called upon to do so.

5. To organise a scheme of personal canvass of all non-panel doctors, with a view to bringing them into the National Medical Union.

### Sussex.

1. That the National Insurance Acts of 1911 and 1913 should be repealed and that a voluntary system be established.

2. That whether the Acts be repealed or amended the conditions of medical service should be rearranged and made more acceptable to the profession.

### Crystal Palace.

1. To promote the union of non-panel practitioners.

2. To further the interests of non-panel practitioners.

3. To promote legislation to secure (*a*) Adequate remuneration; (*b*) Effective medical representation on Insurance Committees; (*c*) Disciplinary control of the medical service entirely in the hands of a medical committee; (*d*) Voluntary health insurance; (*e*) Free choice of doctor.

4. To oppose any prospective legislation to include dependants of insured persons participating in the medical benefits of the Act.

5. To promote the payment of sanatorium and sick benefits on non-panel practitioners' certificates.

### Hampstead.

1. The immediate formation of a practical positive policy by the Council of the National Medical Union with regard to the National Insurance Acts is necessary to increase the membership of the Union.

2. The policy of the National Medical Union, with regard to the National Insurance Acts, should be to obtain such amendments in the administration of medical benefit, and in the enactments relating thereto, as shall give effect to the principle that every insured person making application individually shall be allowed to make his own arrangements for obtaining medical attendance and treatment, without restrictions; and where any contract is required it shall be in the nature of a private contract between patient and private practitioner, and no contract shall be signed between the doctor and Commissioners or any Committee under the National Insurance Acts. Provided always that the scale of fees or charges be not less than those authorised under the existing panel conditions.

3. The Council should proceed to appoint a Parliamentary Sub-Committee, whose duties shall include lobbying, formulating questions for Parliament, approaching possible candidates at elections, and doing everything possible to bring the policy of the Union before Parliament.

4. A Committee consisting of the London members of the Council, with such other London members of the Union as may be co-opted from time to time, should be appointed, with power to act at its discretion in all matters appertaining to the Metropolitan area.

5. The National Medical Union should protest against the distribution of any unallotted medical benefit funds amongst panel doctors until inquiry has been made amongst all the insured persons who have not selected a panel doctor, to ascertain how many of them (*a*) have been attended since the Act by non-panel doctors during illness, or (*b*) intend, if illness occurs, to be so attended. The sums due on account of medical benefit to all such persons should be placed in an "Own Arrangements" Fund and used for defraying, as far as such funds go, the medical expenses of these persons.

### Islington and St. Pancras.

The policy of the Islington and St. Pancras Association is practically one of *no contract*. That is: they are in favour of the patient having free choice of doctor and power to claim refund of the amount of his medical benefit; this was allowed in Section 15 (3) of the Act of 1911. They are also in favour of as much local autonomy as is consistent with union.

### Wandsworth.

1. To organise the medical profession to the highest possible degree of efficiency in defence of its freedom and protection of its economic interests.

2. To lay down the principle of unrestricted free choice of doctor in any form of National Insurance, and to insist upon the unrestricted right of insured persons to make their own arrangements in connection with the Insurance Acts, 1911-1913.

3. Repeal of the medical benefit section of the Insurance Acts should be incorporated in the policy of the National Medical Union.

4. The payment of Insurance premiums should be made collectively by the Insurance Authority to Associations of doctors, by whom the sum total should be divided on the basis of payment for work done.

### Willesden.

1. That a clear statement in general terms be made of the demands of non-panel practitioners and that an equally clear statement be made of what non-panel practitioners have to offer to the public, such as: (*a*) Endeavouring to secure to non-panel practitioners those insured persons who wish to be attended by them, by means of representations or deputations to the Insurance Commissioners or Insurance Committees as to the conditions of "contracting out." (*b*) Making it known that membership of the National Medical Union implies that the interest of the patient shall be considered before the personal advantage of the practitioner, but that the practitioner shall refuse to give his services without a reasonable fee.

2. That the National Medical Union, while being politically non-party, shall endeavour to further the interests of non-panel practitioners by representations to all political parties.

### Meeting of London Organisation Committee.

At a meeting of the Organisation Committee for London on March 9, the following resolution was unanimously passed: "That the London Organisation Committee of the National Medical Union repudiates the action of the Metropolitan Counties Branch of the British Medical Association in attempting to elect a Local Medical Committee for the County of London which would fail to be representative of the practitioners of the area and which would be especially prejudicial to the interests of non-panel practitioners."

The Secretaries were instructed to forward the above resolution to the Council of the British Medical Association and to the Council of the Metropolitan Counties Branch.

They were also instructed to circularise the non-panel profession in London, recommending all non-panel practitioners to abstain from nominating, being nominated, or taking any part whatever in the above election.

### Annual Meeting of Hampstead Non-Panel Associatoin.

The annual meeting of the Hampstead Non-Panel Association was held on Friday, March 6, at the Hampstead Conservatoire. Dr. J. Ford Anderson was in the chair. The total membership at the present time is fifty-nine. There have been, since the decision of the Association to federate under the National Medical Union in December last, fourteen resignations and nine new members. The present officers and committee were reappointed, with power to add additional members. It was decided to hold four general meetings a year, at three of which discussions on medico-ethical and political subjects shall be arranged.

The resolutions mentioned above, embodying recommendations for the policy of the National Medical Union, were unanimously passed. The attempt of the Metropolitan Counties Branch of the British Medical Association to hold an election to appoint a Medical Committee for London under the Insurance Acts was discussed, and it was resolved that this Association is of opinion that it is inadvisable for the members of the National Medical Union to take part in this election, but that, whatever action the Union agrees upon, the London members of the Union be circularised in order to obtain unanimous action. It was resolved to send a letter thanking Mr. Worthington Evans for the yeoman services he has rendered since the introduction of the Insurance Acts, and to enclose a copy of the above resolutions on policy.

### Obituary.

The National Medical Union deeply regrets to have to record the death of one of its prominent members, Dr. J. E. O'Sullivan, of Liverpool, on Thursday, March 5. Dr. O'Sullivan was a Justice of the Peace, and exercised judicial authority in that capacity under the Lunacy Acts. He was an Edinburgh licentiate and assistant physician to the Catholic Blind Asylum.

